# Human Papillomavirus Genotype Distribution among Cervical Cancer Patients prior to Brazilian National HPV Immunization Program

**DOI:** 10.1155/2017/1645074

**Published:** 2017-04-23

**Authors:** Liz M. de Almeida, Luís Felipe L. Martins, Valéria B. Pontes, Flávia M. Corrêa, Raquel C. Montenegro, Laine C. Pinto, Bruno M. Soares, João Paulo C. B. Vidal, Shayany P. Félix, Neilane Bertoni, Moysés Szklo, Miguel Angelo M. Moreira

**Affiliations:** ^1^Division of Population Research, National Cancer Institute, Rio de Janeiro, RJ, Brazil; ^2^Department of Woman Health, Faculty of Medicine, Federal University of Pará, Belém, PA, Brazil; ^3^Genetic Department, National Cancer Institute, Rio de Janeiro, RJ, Brazil; ^4^Epidemiology Department, Johns Hopkins Bloomberg School of Public Health (JHU), Baltimore, MD, USA

## Abstract

To evaluate the impact of HPV immunization and possible changes in virus type-specific prevalence associated with cervical cancer, it is important to obtain baseline information based on socioeconomic, educational, and environmental characteristics in human populations. We describe these characteristics and the type-specific HPV distribution in 1,183 women diagnosed with cervical cancer in two Brazilian healthcare institutions located at the Southeastern (Rio de Janeiro/RJ) and the Amazonian (Belém/PA) regions. Large differences were observed between women in these regions regarding economic, educational, and reproductive characteristics. The eight most frequent HPV types found in tumor samples were the following: 16, 18, 31, 33, 35, 45, 52, and 58. Some HPV types classified as unknown or low risk were found in tumor samples with single infections, HPV 83 in RJ and HPV 11, 61, and 69 in PA. The proportion of squamous cervical cancer was lower in RJ than in PA (76.3% versus 87.3%, *p* < 0.001). Adenocarcinoma was more frequent in RJ than in PA (13.5% versus 6.9%, *p* < 0.001). The frequency of HPV 16 in PA was higher in younger women (*p* < 0.05). The success of a cervical cancer control program should consider HPV types, local health system organization, and sociodemographic diversity of Brazilian regions.

## 1. Introduction

Cervical cancer is the fourth most common neoplasm among women worldwide, with the majority of cases occurring in developing countries, and it is responsible for more than 260,000 yearly deaths [1]. Human papillomavirus (HPV) is a necessary cause of cervical cancer. Twelve HPV types are classified as “high risk” (HPV: 16, 18, 31, 33, 35, 39, 45, 51, 52, 56, 58, and 59), and eight are probably (HPV 68) or possibly oncogenic (HPV: 26, 53, 66, 67, 70, 73, and 82). HPV 16 and 18 are the most prevalent types in cervical tumor samples worldwide, being identified in 60–80% of cases [2, 3]. Prevalence of the other types varies across countries and continents [4, 5].

Early detection of precancerous lesions using the Pap test is the main population-based screening strategy. As the Pap test is only one of the steps in the screening process, this strategy requires the existence of an efficient health system and, thus, its implementation has been more successful in developed than in developing countries. More specifically, after the detection of a cytological cervical precursor lesion (high-grade squamous intraepithelial lesion (HSIL) or adenocarcinoma in situ (AIS)), it is necessary to confirm the diagnosis and to deliver treatment, which involves full access to different facilities of the health system [6]. The absence of such system explains why, in developing countries, cervical cancer mortality has been slow to decline [7].

With the advent of first- and second-generation type-specific HPV vaccines targeting the most prevalent carcinogenic types (16, 18, 31, 33, 45, 52, and 58), a new preventive paradigm has emerged. However, the impact of this strategy on mortality rates will be observed only in the future, and its effectiveness depends on the coverage of these vaccines in the target population [8].

In Brazil, in 2016, cervical cancer incidence rate was estimated at 15.85/100,000 women, representing the 3rd most incident cancer for this group. Incidence rates, however, vary markedly by region. Cervical cancer is the most common female cancer in the Northern (Amazonian) region, with an incidence of about 23.97/100,000 women, which is about twice as high as the incidence in the Southeastern region (11.30/100,000 women) [9, 10].

Age-adjusted cervical cancer mortality in Brazil in 2014 was 4.88/100,000 women, representing the 4th most lethal cancer. The variation in mortality for region is even more striking than that for incidence: it was 11.75/100,000 in the Northern region (ranking the first cause of mortality) and 3.47/100,000 for Southeast region (sixth cause), that is, greater than a 3-fold difference [9, 10]. Variability in the access to information, prevention, diagnosis, and treatment in the healthcare system, as well as educational and economic factors, can explain part of this heterogeneity across regions [11].

Brazil started to implement the Cervical Cancer Control Program in its Public Health System in 1996, based on the Pap test. This program has increased its coverage in the whole country, since 2002. In 2013, a population-wide survey showed that 79.4% of women between the ages of 25 and 64 years (target population) reported at least one Pap's smear test in the past three years [12]. In the Northern region, this proportion was 75.5%; in the Southeastern region, it was 81.1%. In 2014, the National HPV Immunization Program started to use the 4-valent vaccine (HPV 6, 11, 16, and 18) for girls between the ages of 9 and 13 years, in three doses, at 0, 6, and 60 months. In 2015, this schedule changed to two doses, at 0 and 6 months.

In the present paper we describe the sociodemographic profile and type-specific HPV distribution by histological tumor type of women diagnosed with cervical cancer in reference public hospitals in two Brazilian capitals: Rio de Janeiro city (Rio de Janeiro State), in the Southeastern region, and Belém city (Pará State), in the Northern (Amazonian) region. These institutions receive the majority of the cervical cancer cases diagnosed in their respective States. The areas have distinct socioeconomic characteristics expressed by the Human Developmental Index (HDI): 0.761 for Rio de Janeiro and 0.646 for Pará. The principal aim of this study is to provide baseline information of type-specific HPV frequencies in women with cervical cancer in these distinct scenarios, for future evaluation after the implementation of the HPV immunization program in the country.

## 2. Methods

### 2.1. Patients and Data Collection

Participants were patients with newly diagnosed cervical cancer at the Brazilian National Cancer Institute (in Portuguese: Instituto Nacional de Câncer, INCA) in Rio de Janeiro (RJ) and Hospital Ophir Loyola (HOL) in Belém (PA). Between August 2012 and March 2014 in INCA and from April 2013 to September 2015, all the patients who attended the hospitals for the first clinical visit were invited to participate of the study.

Included in the study were women with histopathological diagnosis of cervical cancer, aged ≥ 18 years, with no previous oncological treatment for cervical cancer (i.e., cancer surgery, radiotherapy, or chemotherapy) and those who had performed a biopsy during this first clinical visit. Women with cognitive or physical abnormalities that prevented them from answering the questionnaire were excluded from the study.

A pretested questionnaire was used to collect data on socioeconomic variables, knowledge about cervical cancer prevention, access to diagnosis and treatment, hormonal and reproductive histories, and tobacco use. The criteria for definitions of sociodemographic variables in this study were those used by the Brazilian Geographic and Statistics Institute (IBGE). Information on histological diagnosis and tumor stage was collected from medical records. The sample for type identification was collected by a gynecologist (*ca*. 5 mm in all dimensions) during the clinical visit, using a forceps, and stored in RNA-Latter until nucleic acid isolation.

Interviews were conducted by trained research nurses, who were also responsible for storage, labeling, and sending biopsied specimens to the research laboratories. “Over-the-shoulder” monitoring of interviewers, review of instruments, and laboratory visits were conducted every two months by the central coordinator group (INCA). If problems were noted, the group met with the data collectors and technicians for problem solving.

The Institutional Ethic Committees approved all the procedures and all patients signed an informed consent form (INCA (RJ): CEP 156/10 and CAAE: 53398416.0.0000.5274, in 02/25/2011 and, in HOL (PA): CAAE: 03288212.0.1001.0018, in 11/28/2012).

### 2.2. DNA Isolation and HPV Identification

DNA was isolated from biopsies using the QIAamp DNA Mini Kit (Qiagen®). HPV detection was carried out by PCR amplification using primer sets PGMY07 and PGMY09 [13] and reactions without PCR products were submitted to a nested PCR with the primers GP5+/GP6+ [14]. Genomic DNA from CasKi and Hela cell lines were used as positive controls for PCR reaction. Samples negative for HPV DNA amplification after nested PCR were subjected to a PCR reaction for *β*-globin, and positive reactions to *β*-globin and negative ones for nested PCR were considered negative for HPV. For HPV identification, PCR products were purified with Illustra GFX PCR DNA and Gel Band Purification Kit (GE Healthcare®) and subjected to DNA sequencing in both directions, using the Big Dye Terminator Cycle Sequencing Ready Reaction V3.1 Kit (Life technologies®), following the supplier's instructions.

The electropherograms of each sample were checked by eye and a consensus sequence of the bidirectional sequencing was subjected to HPV type identification using the Blast software [15]. Samples with electropherograms showing overlapping sequence peaks were considered as potentially coinfected by multiple HPV types. These samples were evaluated with the High + Low Papillomastrip Kit (OPERON®) following the instructions of the supplier. This Kit allows the identification of 37 HPV types, 6, 11, 16, 18, 26, 31, 33, 35, 39, 40, 42, 43, 44, 45, 51, 52, 53, 54, 56, 58, 59, 61, 62, 66, 67, 68, 69, 70, 71, 72, 73, 74, 81, 82, 83, 84, and 91. Samples with electropherograms showing very low peak fluorescence were considered as HPV type not identified (or HPV-X).

### 2.3. Storage and Data Analysis

Epidemiological data were stored using Epi-Info software and then linked to both the clinical data and the DNA HPV data. The final database was analyzed using Stata v.12.0. Categorical variables of women's characteristics were reported as counts and percentages and compared between cities using Chi-squared or Fisher's exact tests.

## 3. Results

A total of 1,702 women (968 in RJ and 734 in PA) with cervical cancer diagnosis signed the informed consent and were interviewed, but 1,198 (70.4%) were biopsied. The reasons for not being biopsied were technical difficulties related to sample collection (271), clinical conditions of the patient (86), and refusal (147). Of the 1,198 samples, 11 were excluded due technical problems in the handling of the sample, and the HPV DNA was not detected in four samples by nested PCR using the primers PGMY and GP5+/6+. Thus, the final sample of this study is 1,183 women (590 from RJ and 593 from PA).

### 3.1. Socioeconomic Characteristics

Characteristics of participants are shown in Table 1. The majority of the 1,183 women were not residents in the cities where the hospitals were located, and this proportion was higher in Pará than in Rio de Janeiro. Age distributions were similar in both institutions. About 1/4 of the women in RJ had less than three years of schooling and this proportion was almost 50% in PA. According to the interviewers' observation, mixed race was predominant in both cities but the proportions were different between cities (53.4 in RJ and 82.3% in PA). The majority of women in both sites reported to have no paid work, and the proportions of women with an average per capita household income less than 1/2 minimum wage (≈US$ 160) were twice as high in PA as in RJ.

### 3.2. Sexual Behaviors and Reproductive History

The majority of women initiated sexual activity between 16 and 18 years old (about 40% in both sites), and the proportions of first sexual intercourse before age 16 years were lower in RJ than in PA. The proportion of women who reported more than five lifetime sexual partners was much higher in RJ than in PA. Women with one or two childbirths predominated in RJ, while in PA women with seven or more childbirths were most often represented (Table 2).

### 3.3. Knowledge and Preventive Practices

Knowledge of the Pap test's purpose was lower in PA (31.9%) than in RJ (60.1%). Around 80% of women in RJ and 75% in PA had had at least one Pap test before the current health problem. Frequency of Pap smear test was also different between States, and about half of patients from RJ reported to never being tested before or having the test with an interval longer than 3 years, but in PA the proportions were around 80% (Table 2).

Besides, the frequency of never smokers was about the same in both locations, current smoker proportion was higher in Rio de Janeiro than in Pará (Table 2).

### 3.4. HPV Genotypes

We identified 23 HPV types in Pará and 19 HPV types in Rio de Janeiro, considering tumors with both single and multiple HPV infection. The seven oncogenic HPV types with the highest frequencies in RJ were 16, 18, 45, 35, 31/33, and 58, while in PA they were 16, 18, 33/45, 31, 52, and 35.

Of the 1183 women, the sequence analysis identified 1114 women with single infection (554 from RJ and 558 from PA), corresponding to 96.6% and 95.4%, respectively. HPV genotype was not identified in 22 cases (HPV-X). In tumor samples with single infections, from those HPV types considered as “unknown or low risk group,” we found only one type in RJ (HPV 83) and three types in PA (HPV 11, 61, and 69) (Table 3).

Histological tumor type distribution of woman with single infection varied across sites. The proportion of squamous cervical cancer (SCC) was lower in RJ than in PA (76.3% versus 87.3%, *p* < 0.001). Adenocarcinoma (ADC) represented 13.5% of tumors in RJ and 6.9% in PA (*p* < 0.001) (Table 4).

Multiple infections were identified in 47 women (20 from RJ and 27 from PA). HPV 16 was found in 40 of the 41 tumors and HPV 18 in 25 of the 41 tumors. In only one case of multiple infections HPV 16 was not present. In six samples it was not possible to identify the HPV type due to the small amount of DNA. The “unknown or low risk” HPV 42 and 54 types appeared in association with high-risk types (see Supplementary Table  1 in Supplementary Material available online at https://doi.org/10.1155/2017/1645074).

The proportions of HPV 16, 18, Alpha-7, and Alpha-9 by age and location are shown in Supplementary Table  2. With the exception of Pará, where HPV 16 seems to be more common in the younger age groups (*p* < 0.05), these proportions were fairly similar to the other types in both States.

## 4. Discussion

The two Brazilian States where the study was carried out have distinct socioeconomic characteristics. Rio de Janeiro has the second economy in Brazil. With an estimated population for 2015 of 16,550,024 distributed in 92 municipalities, the population density is 365.23 per km^2^ (estimated for 2010) and the household monthly nominal income per capita in 2015 was R$1,285 (~US$390). Pará has the third-worst Human Development Index (HDI) among the Brazilian States [16]. It has an estimated population of 8,175,113 located in 144 municipalities [17], with a population density estimated in 6.07 per Km^2^. The nominal household monthly income per capita of the resident population was R$672 (~US$202) [16].

These characteristics are reflected in our findings with regard to the economic, educational, and behavioral/sexual differences observed between the two groups of women. These distinct characteristics may also reflect the differences observed in cervical cancer incidence rate for 2016 (16.9 per 100,000 for RJ and 20.52 per 100,000 for PA) and in the age-adjusted cervical cancer mortality rate for 2014 (8.60 per 100,000 for the PA and 4.75 per 100,000 for the RJ) [10]. Difficulties in access to the centers of diagnosis and treatment of cervical precancerous lesions in the Amazonian Region are probably a major factor resulting in its high mortality.

Alpha-7 HPV types (HPV 18, 39, and 45) were more frequent in RJ than in PA. As they are more strongly associated with adenocarcinoma, it may explain the difference observed in distribution of histological types of tumors between the Brazilian States. A growing body of evidence suggests that cervical cancer screening is less effective against adenocarcinoma than squamous carcinoma [18]. As a result, squamous carcinoma incidence has declined in countries with well-organized screening programs, and adenocarcinoma has become relatively more common. Therefore the difference in adenocarcinoma proportion observed between the cities in this report may probably be due to disparate screening effectiveness.

Of the probably or possibly carcinogenic types, the most common type in our study was HPV 73, thus suggesting that its carcinogenic potential should be further evaluated in future studies.

The lower diversity of HPV types, in RJ than in PA (23 and 19 HVP types, resp.), was also observed in another Southeastern State, São Paulo [19], where 17 different HPV types were identified by Linear Array HPV Genotyping Test (LA, Roche Molecular Diagnostic). Comparisons with other Brazilian data from cervical cancer samples are limited due to either the techniques used for HPV identification (which only allowed identification of a few HPV types other than HPV16 and HPV18) or the small number of samples analyzed [20–24].

A comparison of our data on HPV type frequency with those from de Oliveira et al. [19] shows that in PA, RJ, and São Paulo States, HPV 16, HPV 18, HPV 31, HPV 33, and HPV 45 are among the seven most frequent HPV types, with HPV 35 also included in São Paulo and RJ. In PA, HPV 52 is the sixth (2.7%) and HPV 35 is the seventh most frequent type (2.5%). All high-risk HPV types present in the 9-valent vaccine (16, 1, 31, 33, 45, 52, and 58, Merck & Co. Inc., Whitehouse Station, NJ, USA) are among the seven most frequent HPV types in these States.

The frequency of multiple HPV infections detected here (4.1%) was lower than that reported in other studies in Brazil, 9%, by Fernandes et al., 2010 [23] and 24%, by de Oliveira et al., 2013 [19], although Eluf-Neto et al. (1994) [20] found similar frequency (4.3%). These authors used different strategies (based on PCR and DNA hybridization) to identify the viruses in single and multiple infections.

One of the limitations of our study is that the methodology to detect multiple infections, based on DNA sequencing (using the Sanger method), does not detect less frequent HPV DNA in a tumor sample (<20%) [25]. In addition, the* High + Low Papillomastrip Kit* (OPERON) only allows the identification of specific HPV types for which there are probes in the Kit.

Considering the results obtained in this study, the 4-valent HPV vaccine, adopted by the Public Health System in Brazil since 2014, can potentially reduce at least 70% of cervical cancer incidence in RJ and PA. Implementation of the new 9-valent HPV vaccine has the potential of increasing the impact of vaccination on cervical cancer incidence by 85%. However, one of the nine most frequent carcinogenic types, HPV 35, has not been included in the current available vaccines, a limitation that may impact on HPV primary prevention in Brazil.

In addition to vaccination, the policy for Cervical Cancer Control in Brazil recommends the maintenance of screening programs for all the women at the age of 25 through 64 years old. The success of this strategy to control cervical cancer has to take into account the distinct demographic and sociocultural characteristics of the different Brazilian regions. Given its lower population density and transportation problems, the Amazonian Region poses a special challenge with regard to access of its population to the healthcare system.

## Supplementary Material

Multiple infections were identified in 47 women (20 from RJ and 27 from PA). HPV 16 was found in 40 of the 41 tumors and HPV 18 in 25 of the 41 tumors. In only one case of multiple infections HPV 16 was not present. In six samples it was not possible to identify the HPV type due to the small amount of DNA. The “unknown or low risk” HPV 42 and 54 types appeared in association with high-risk types.

## Figures and Tables

**Table 1:** Sociodemographic characteristics of women with cervical cancer in Rio de Janeiro and Pará, Brazil.

Sociodemographic characteristic	Rio de Janeiro (*n* = 590)		Pará (*n* = 593)		*p* value^a^
	*n*	%	*n*	%	
*City of residence*					
State's capital	217	36.8	167	28.2	0.002
Other cities	373	63.2	426	71.8	
*Age (years)*					
18 to 39	160	27.1	146	24.6	0.315
40 to 49	163	27.6	165	27.8	
50 to 64	191	32.4	184	31.0	
65 and older	76	12.9	98	16.6	
*Education (years of schooling)*					
None	43	7.3	133	22.4	<0.001
1 to 3	112	19.0	148	25.0	
4 to 7	180	30.5	164	27.6	
8 to 10	134	22.7	77	13.0	
11 and more	121	20.5	71	12.0	
*Marital status*					
Single	47	8.0	28	4.7	0.007
Married/stable union	340	57.6	320	54.0	
Divorced/separated	121	20.5	165	27.8	
Widow	82	13.9	80	13.5	
*Religion*					
None	32	5.4	6	1.0	<0.001
Catholic	279	47.3	341	57.5	
Protestant	248	42.0	243	41.0	
Others	31	5.3	3	0.5	
*Race/skin color*					
White	197	33.4	70	11.8	<0.001
Mixed	315	53.4	488	82.3	
Black	76	12.9	26	4.4	
Others	2	0.3	9	1.5	
*Paid work*					
No	355	60.2	469	79.1	<0.001
Yes	235	39.8	124	20.9	
*Average household income per capita*					
1/2 minimum wage or less	178	35.7	271	72.3	<0.001
1/2 to 1 minimum wage	173	34.7	85	22.7	
More than 1 minimum wage	148	30.6	19	5.0	

*Note*. % based on valid information.

^a^Chi-squared or Fisher Exact test.

**Table 2:** Characteristics and behaviors of women with cervical cancer in Rio de Janeiro and Pará, Brazil.

Characteristic	Rio de Janeiro (*n* = 590)		Pará (*n* = 593)		*p* value^a^
	*n*	%	*n*	%	
*Sexual activity onset*					
8–15 years old	165	28.9	211	39.5	<0.001
16–18 years old	227	39.7	218	40.8	
19 years or older	179	31.4	105	19.7	
*Lifetime number of sexual partners*					
1-2	204	36.7	245	50.6	<0.001
3–5	253	45.5	203	41.9	
6 or more	99	17.8	36	7.5	
*Number of childbirths*					
None	11	1.9	5	0.9	<0.001
1-2	233	41.4	102	17.5	
3-4	189	33.6	178	30.5	
5-6	84	14.9	113	19.3	
7 or more	46	8.2	186	31.8	
*Knowledge of Pap test's purpose*					
Yes	355	60.2	189	31.9	<0.001
No	235	39.8	404	68.1	
*Pap test performed before the current health problem*					
Yes	477	80.8	426	74.1	0.006
No	113	19.2	149	25.9	
*Frequency of Pap test*					
None	113	19.2	149	26.0	<0.001
Annually	212	36.0	108	18.8	
Every two years	48	8.2	11	1.9	
Every three years	7	1.2	1	0.2	
Interval longer than three years or irregular testing	209	35.4	305	53.1	
*Tobacco use status*					
Current smoker	114	19.3	68	11.5	<0.001
Former smoker	209	35.4	260	43.8	
Never smoker	267	45.3	265	44.7	

*Note*. % based on valid information.

^a^Chi-squared or Fisher's exact test.

**Table 3:** Distribution of HPV genotypes, according to infection type, in tumor samples of women with cervical cancer in Rio de Janeiro and Pará, Brazil.

HPV type by risk group	Single infection					Multiple infection					Total				
	Rio de Janeiro		Pará			Rio de Janeiro		Pará			Rio de Janeiro		Pará		
	*n*	%	*n*	%		*n*	%	*n*	%		*n*	%	*n*	%	
*Carcinogenic (high risk)*															
16	373	65.4	371	65.5		19	29.7	27	36.0		392	61.8	398	62.1	
18	76	13.3	58	10.2	†	13	20.3	12	16.0		89	14.0	70	10.9	†
31	7	1.2	16	2.8	*∗*	6	9.4	3	4.0		13	2.1	19	3.0	
33	7	1.2	16	2.8	*∗*	6	9.4	10	13.3		13	2.1	26	4.1	*∗*
35	12	2.1	14	2.5		2	3.1	2	2.7		14	2.2	16	2.5	
39	6	1.1	5	0.9		4	6.3	0	0.0	-	10	1.6	5	0.8	
45	33	5.8	20	3.5	†	5	7.8	6	8.0		38	6.0	26	4.1	
51	2	0.4	5	0.9		0	0.0	0	0.0	-	2	0.3	5	0.8	
52	8	1.4	14	2.5		2	3.1	3	4.0		10	1.6	17	2.7	
56	2	0.4	3	0.5		0	0.0	1	1.3	-	2	0.3	4	0.6	
58	11	1.9	6	1.1		1	1.6	3	4.0		12	1.9	9	1.4	
59	6	1.1	9	1.6		0	0.0	0	0.0	-	6	0.9	9	1.4	

*Probably, possibly carcinogenic*															
26	2	0.4	3	0.5		0	0.0	0	0.0	-	2	0.3	3	0.5	
53	0	0.0	1	0.2	-	0	0.0	0	0.0	-	0	0.0	1	0.2	-
66	0	0.0	1	0.2	-	0	0.0	0	0.0	-	0	0.0	1	0.2	-
67	0	0.0	1	0.2	-	0	0.0	1	1.3	-	0	0.0	2	0.3	-
68	2	0.4	1	0.2		1	1.6	0	0.0	-	3	0.5	1	0.2	
70	0	0.0	2	0.4	-	0	0.0	0	0.0	-	0	0.0	2	0.3	-
73	8	1.4	5	0.9		0	0.0	0	0.0	-	8	1.3	5	0.8	
82	0	0.0	1	0.2	-	0	0.0	1	1.3	-	0	0.0	2	0.3	-

*Unknown or low risk*															
11	0	0.0	1	0.2	-	0	0.0	0	0.0	-	0	0.0	1	0.2	-
42	0	0.0	0	0.0	-	1	1.6	0	0.0	-	1	0.2	0	0.0	-
54	0	0.0	0	0.0	-	3	4.7	0	0.0	-	3	0.5	0	0.0	-
61	0	0.0	1	0.2	-	1	1.6	0	0.0	-	1	0.2	1	0.2	
69	0	0.0	4	0.7	-	0	0.0	0	0.0	-	0	0.0	4	0.6	-
83	1	0.2	0	0.0	-	0	0.0	0	0.0	-	1	0.2	0	0.0	-

*Other*															
*X* ^≈^	14	2.5	8	1.4		0	0.0	6	8.0		14	2.2	14	2.2	

*Total*	*570*		*566*			*64*		*75*			*634*		*641*		

^≈^ *X*: HPV DNA not identified.

Comparison of HPV proportion between sites: ^*∗*^*p* value < 0.05; ^†^*p* value < 0.10; ^−^test not performed.

**Table 4:** Distribution of HPV genotypes, according to histological tumor type, in tumor samples of women with single infection in Rio de Janeiro and Pará, Brazil.

	SCC^+^					ADC^+⁡+^					Other				
HPV type by risk group	Rio de Janeiro		Pará			Rio de Janeiro		Pará			Rio de Janeiro		Pará		
	*n*	%	*n*	%		*n*	%	*n*	%		*n*	%	*n*	%	
*Carcinogenic (high risk)*															
16	296	68.0	324	65.6		45	58.4	25	64.1		32	55.2	22	66.7	
18	38	8.7	42	8.5		22	28.6	10	25.6		16	27.6	6	18.2	
31	7	1.6	16	3.2		0	0.0	0	0.0	-	0	0.0	0	0.0	-
33	7	1.6	16	3.2		0	0.0	0	0.0	-	0	0.0	0	0.0	-
35	9	2.1	14	2.8		2	2.6	0	0.0	-	1	1.7	0	0.0	-
39	5	1.1	5	1.0		0	0.0	0	0.0	-	1	1.7	0	0.0	-
45	24	5.5	15	3.0	†	6	7.8	3	7.7		3	5.2	2	6.1	
51	2	0.5	4	0.8		0	0.0	0	0.0	-	0	0.0	1	3.0	-
52	8	1.8	14	2.8		0	0.0	0	0.0	-	0	0.0	0	0.0	-
56	2	0.5	2	0.4		0	0.0	0	0.0	-	0	0.0	1	3.0	-
58	11	2.5	6	1.2		0	0.0	0	0.0	-	0	0.0	0	0.0	-
59	4	0.9	8	1.6		1	1.3	1	2.6		1	1.7	0	0.0	-

*Probably, possibly carcinogenic*															
26	2	0.5	3	0.6		0	0.0	0	0.0	-	0	0.0	0	0.0	-
53	0	0.0	1	0.2	-	0	0.0	0	0.0	-	0	0.0	0	0.0	-
66	0	0.0	1	0.2	-	0	0.0	0	0.0	-	0	0.0	0	0.0	-
67	0	0.0	1	0.2	-	0	0.0	0	0.0	-	0	0.0	0	0.0	-
68	2	0.5	1	0.2		0	0.0	0	0.0	-	0	0.0	0	0.0	-
70	0	0.0	2	0.4	-	0	0.0	0	0.0	-	0	0.0	0	0.0	-
73	7	1.6	5	1.0		0	0.0	0	0.0	-	1	1.7	0	0.0	-
82	0	0.0	1	0.2	-	0	0.0	0	0.0	-	0	0.0	0	0.0	-

*Unknown or low risk*															
11	0	0.0	1	0.2	-	0	0.0	0	0.0	-	0	0.0	0	0.0	-
61	0	0.0	1	0.2	-	0	0.0	0	0.0	-	0	0.0	0	0.0	-
69	0	0.0	4	0.8	-	0	0.0	0	0.0	-	0	0.0	0	0.0	-
83	1	0.2	0	0.0	-	0	0.0	0	0.0	-	0	0.0	0	0.0	-

*Other*															
*X* ^≈^	10	2.3	7	1.4		1	1.3	0	0.0	-	3	5.2	1	3.0	

*Total*	*435*		*494*			*77*		*39*			*58*		*33*		

^+^SCC: Squamous cervical cancer. ^++^ADN: Adenocarcinoma.

^≈^ *X*: HPV DNA not identified.

Comparison of HPV proportion between sites: ^*∗*^*p* value < 0.05; ^†^*p* value < 0.10; ^−^test not performed.
