# Supplementary material for: Human Papillomavirus Genotype Distribution among Cervical Cancer Patients prior to Brazilian National HPV Immunization Program
Source: J Environ Public Health. 2017 Apr 23;2017:1645074. doi: 10.1155/2017/1645074 (PMC5420420; doi:10.1155/2017/1645074)
Supplement: Supplementary file 1 — Multiple infections were identified in 47 women (20 from RJ and 27 from PA). HPV 16 was found in 40 of the 41 tumors and HPV 18 in 25 of the 41 tumors. In only one case of multiple infections HPV 16 was not present. In six samples it was not possible to identify the HPV type due to the small amount of DNA. The “unknown or low risk” HPV 42 and 54 types appeared in association with high-risk types. [file 1645074.f1.pdf]

Supplementary Table 1 – Distribution of HPV type-specific combinations found in multiple infections in tumor samples of women with cervical cancer in Rio de Janeiro and Pará, Brazil

HPV types in multiple infections	Rio de Janeiro	Pará
HPV16, HPV18	4	3
HPV16, HPV33	0	2
HPV16, HPV45	2	2
HPV16, HPV39	1	0
HPV16, HPV42	1	0
HPV16, HPV58	0	1
HPV16, HPV18, HPV31	1	2
HPV16, HPV18, HPV33	0	1
HPV16, HPV18, HPV35	0	1
HPV16, HPV33, HPV45	0	2
HPV31, HPV35, HPV39	1	0
HPV16, HPV18, HPV45	1	0
HPV16, HPV18, HPV52	1	0
HPV16, HPV45, HPV58	0	1
HPV16, HPV18, HPV31, HPV33	1	1
HPV16, HPV18, HPV33, HPV45	1	0
HPV16, HPV18, HPV33, HPV52	0	2
HPV16, HPV18, HPV33, HPV54	1	0
HPV16, HPV18, HPV54, HPV61	1	0
HPV16, HPV18, HPV56, HPV82	0	1
HPV16, HPV33, HPV58, HPV67	0	1
HPV16, HPV45, HPV58, HPV54	1	0
HPV16, HPV18, HPV31, HPV33, HPV39	1	0
HPV16, HPV31, HPV33, HPV39, HPV68	1	0
HPV16, HPV18, HPV31, HPV33, HPV35, HPV52	1	0
HPV16, HPV18, HPV33, HPV35, HPV45, HPV52	0	1
HPV16, *HPV-X**	0	6
**TOTAL**	**20**	**27**

**HPV-X: HPV DNA not identified*

Supplementary Table 2: Frequencies of cases of cervical cancer with a single infection of selected HPV type by age groups and location (Rio de Janeiro and Pará), Brazil

Age (years)	Population in each age group (N)		HPV 16				HPV 18				Alpha-7				Alpha-9			
	Rio de Janeiro	Pará	Rio de Janeiro		Pará		Rio de Janeiro		Pará		Rio de Janeiro		Pará		Rio de Janeiro		Pará	
			n	%	n	%	n	%	n	%	n	%	n	%	n	%	n	%
18 to 34	78	72	54	69.2	51	70.8	10	12.8	9	12.5	15	19.2	14	19.4	59	75.6	56	77.8
35 to 44	148	152	95	64.2	108	71.1	27	18.2	18	11.8	38	25.7	25	16.5	100	67.6	125	82.2
45 to 54	159	145	104	65.4	97	66.9	20	12.6	11	7.6	35	22.0	23	15.9	117	73.6	110	75.9
55 to 64	111	106	72	64.9	67	63.2	12	10.8	10	9.4	23	20.7	15	14.2	85	76.6	82	77.4
65 and older	74	91	48	64.9	48	52.8	7	9.5	10	11.0	10	13.5	17	18.7	57	77.0	66	72.5
p value[a]			0.960		0.042		0.313		0.724		0.326		0.874		0.427		0.481	

Alpha-7: HPV 18, 39, 45, 59, 70, 68, 85. *Alpha-9: HPV 16, 31, 33, 35, 52, 58, 11, 67*

[a] *Chi-squared test*
